# Clinical prognosis of surgical resection versus transarterial chemoembolization for single large hepatocellular carcinoma (≥5 cm): A propensity score matching analysis

**DOI:** 10.1002/kjm2.12640

**Published:** 2023-01-10

**Authors:** Pei‐Min Hsieh, Pojen Hsiao, Yaw‐Sen Chen, Jen‐Hao Yeh, Chao‐Ming Hung, Hung‐Yu Lin, Ching‐Hou Ma, TaoQian Tang, Yu Wei Huang, Pin‐Nan Cheng, Kun‐Chou Hsieh, Kuang‐Chun Hu, Ming‐Jong Bair, Chih‐Wen Lin

**Affiliations:** ^1^ Department of Surgery, E‐Da Hospital I‐Shou University Kaohsiung Taiwan; ^2^ Division of Gastroenterology and Hepatology, Department of Medicine, E‐Da Hospital I‐Shou University Kaohsiung Taiwan; ^3^ Division of Gastroenterology and Hepatology, E‐Da Dachang Hospital I‐Shou University Kaohsiung Taiwan; ^4^ School of Medicine, College of Medicine I‐Shou University Kaohsiung Taiwan; ^5^ Department of Orthopedic Surgery, E‐Da Hospital I‐Shou University Kaohsiung Taiwan; ^6^ Emergency and Critical Care Center, E‐Da Hospital I‐Shou University Kaohsiung Taiwan; ^7^ School of Nursing Fooyin University Pingtung Taiwan; ^8^ Division of Gastroenterology and Hepatology, Department of Internal Medicine, College of Medicine National Cheng Kung University Hospital Tainan Taiwan; ^9^ Healthy Evaluation Center and Division of Gastroenterology, Department of Internal Medicine MacKay Memorial Hospital Taipei Taiwan; ^10^ Mackay Junior College of Medicine, Nursing and Management New Taipei Taiwan; ^11^ Mackay Medical College New Taipei Taiwan; ^12^ Division of Gastroenterology and Hepatology, Department of Internal Medicine Taitung Mackay Memorial Hospital Taitung Taiwan; ^13^ Health Examination Center, E‐Da Hospital I‐Shou University Kaohsiung Taiwan; ^14^ School of Chinese Medicine, College of Chinese Medicine China Medical University Taichung Taiwan; ^15^ Research Center for Traditional Chinese Medicine China Medical University Hospital Taichung Taiwan

**Keywords:** large hepatocellular carcinoma, overall survival, progression‐free survival, surgical resection, transarterial chemoembolization

## Abstract

Favorable prognostic factors and therapeutic strategies are important for patients with single large hepatocellular carcinoma (HCC). This retrospective study aimed to investigate the prognostic factors in patients with single large (≥5 cm) HCC with Child‐Pugh (CP) class A patients and to recommend therapeutic strategies. Overall, 298 HCC patients with single and large (≥5 cm) tumors with CP class A, but without distant metastasis and macrovascular invasion were included, and their clinicopathological data, overall survival (OS), and progression‐free survival (PFS) were recorded. OS and PFS was analyzed by the Kaplan–Meier method and Cox regression analysis. Propensity score matching (PSM) analysis was performed. The 298 HCC patients were 79.2% male and median age of 64 years. For the initial treatment, surgical resection (SR) and transarterial chemoembolization (TACE) was 50.8% and 49.2%, respectively. The OS and PFS were significantly higher in patients receiving SR than those receiving TACE before and after PSM. Furthermore, in multivariate analysis, cirrhosis (Hazard ratio [HR]: 2.04; 95% confidence interval [CI]: 1.35–3.03, *p* < 0.001, CP class A5/6 [HR: 4.01; 95% CI: 2.43–6.66, *p* < 0.001], and initial treatment [SR vs. TACE HR = 3.23; 95% CI: 2.13–5.01, *p* < 0.001]) remained significantly associated with mortality. Moreover, in multivariate analysis, CP class A5/6 (HR: 3.23; 95% CI: 1.89–5.88, *p* < 0.001), and initial treatment (Resection vs. TACE; HR = 4.17; 95% CI: 1.64–8.33, *p* = 0.039) remained significantly associated with recurrence. In conclusion, SR was associated with significantly higher OS and PFS rates than TACE before and after PSM for single large HCC patients.

**Abbreviations**
AASLD: American Association for the Study of Liver Disease
BCLC: Barcelona Clinic Liver Cancer
CI: confidence interval
C‐P class: Child–Pugh class
ESAS: European Association for the Study of Liver
HAIC: hepatic artery infusion chemotherapy
HCC: hepatocellular carcinoma
HR: hazard ratio
OS: overall survival
PFS: progression‐free survival
TACE: transcatheter arterial chemoembolization

## 1 INTRODUCTION

Hepatocellular carcinoma (HCC) is the fifth most commonly occurring cancer and the second most common cause of cancer‐related death worldwide.^1^, ^2^, ^3^, ^4^, ^5^ Although surveillance for HCC with alpha‐fetoprotein (AFP) and ultrasound in patients at risk for HCC is recommended,^3^, ^4^, ^5^ the proportion of diagnosis of large HCC is still high.^6^ Liver resection promoted the long‐term OS in HCC patients across various.

Barcelona clinic liver cancer (BCLC) stages.^7^, ^8^ The BCLC system designated a single large HCC (>5 cm) as BCLC stage A rather than stage B in 2012.^9^ The revised BCLC classification schema has been endorsed by the American Association for the Study of Liver Diseases (AASLD)^3^ and the European Association for the Study of the Liver (EASL).^4^ Moreover, the most recent version of the combined American Joint Committee on Cancer (AJCC)/Union for International Cancer Control (UICC) TNM staging system from 2017 states that patients with multiple tumors, any of which are >5 cm, are categorized as T3.^10^ Recent studies showed that around two‐third of patients with tumor size >10 cm presented microvascular invasion and better outcomes were reported after resection in these patients; Hence, surgical resection should be considered in single large HCC.^6^, ^11^ Several studies also demonstrated that liver resection is safe and effective treatment for single large HCC.^12^, ^13^, ^14^, ^15^, ^16^, ^17^ However, these studies had limitations, such as limited data available, lack of comparison with different treatment, and/or selection bias. Moreover, treatment for single large (≥5 cm) HCCs remains largely unknown and needs to be further studied. This study aimed to investigate the prognostic factors and the effective treatment for single large HCC.

## 2 METHODS

### 2.1 Patients and follow‐up

**FIGURE 1:**
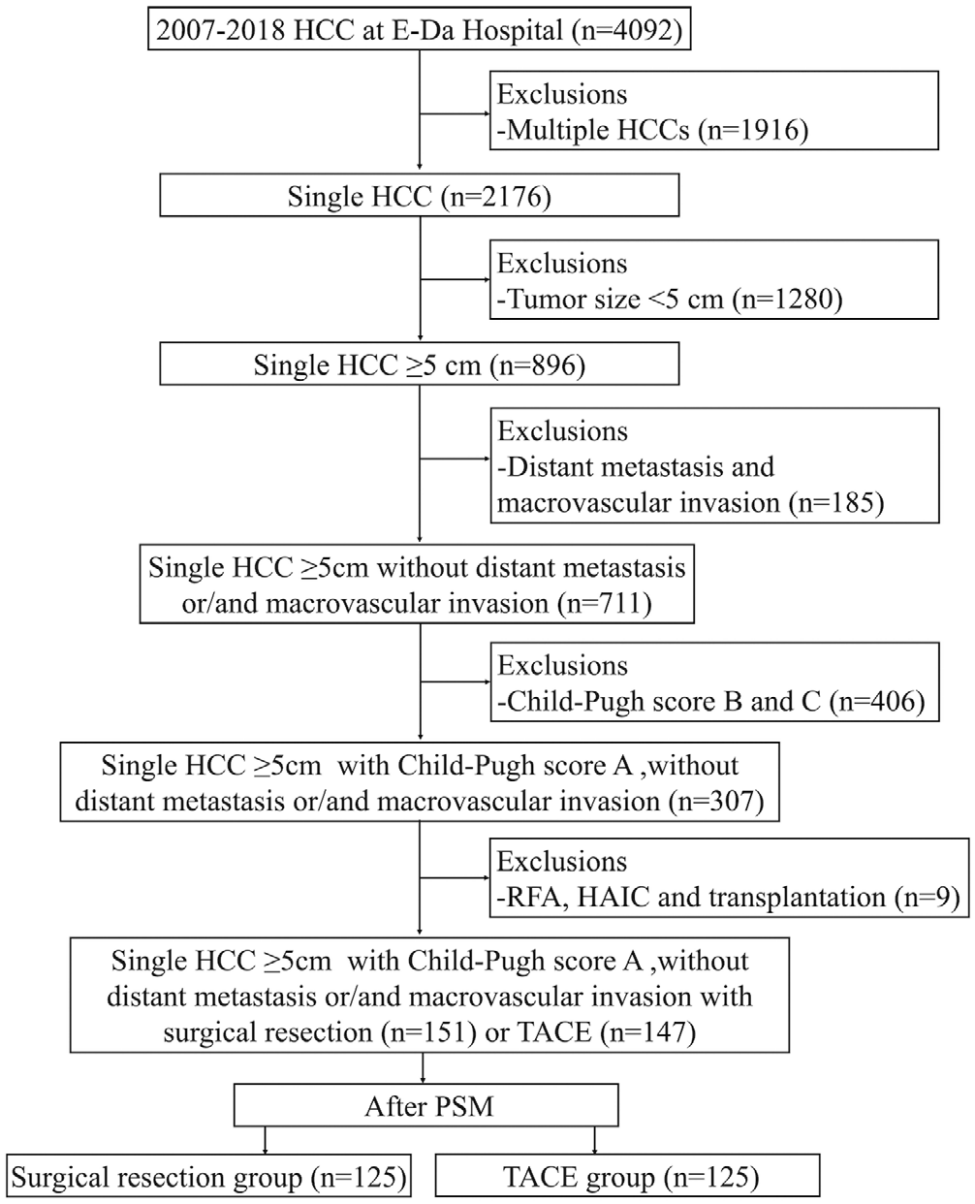
Study flowchart and inclusion of participants.

We retrospectively enrolled clinical data on 4092 patients diagnosed with HCC between 2007 and 2018 at E‐Da Hospital, I‐Shou University, Kaohsiung, Taiwan. Three thousand and seven hundred eight‐seven patients were excluded due to multiple HCC, tumor size <5 cm, the presence of macrovascular invasion or/and distal metastasis and CP class B and C. Finally, 298 HCC patients with single tumor, tumor size ≥5 cm, without macrovascular invasion or/and distal metastasis, and CP class A were included in this retrospective study (Figure 1). This study was approved by the Institutional Review Board at E‐Da Hospital.

Patients were diagnosed with HCC based on histological confirmation or at least one typical imaging method according to the recommendations of the AASLD.^18^

Clinicopathological parameters, including demographic features, smoking, alcohol use, hepatitis status, blood test, liver cirrhosis, Child‐Pugh (CP) class, tumor size, AFP, mortality, disease‐progression, and follow‐up time, were recorded. Tumor number and tumor size were mostly determined based on radiologic findings and confirmed by pathologic findings if appropriate. Liver cirrhosis was diagnosed based on pathologic findings and/or evaluated by ultrasound, computed tomography (CT), or magnetic resonance imaging (MRI). The functional status of the liver was evaluated using the CP class. Patients were treated with surgical resection (SR) or transarterial chemoembolization (TACE). Our multidisciplinary team chose suitable therapy. Patients were followed up every 3–6 months by abdominal ultrasound, CT or MRI and AFP. Overall survival (OS) was defined as the time from the date of diagnosis to the date of death or last visit, and the last follow‐up time was December 2020. Progression‐free survival (PFS) was defined as the time from the date of diagnosis to the date of disease progression or last visit, and the last follow‐up time was December 2020.

### 2.2 Data analysis and statistics

All statistical analyses were performed using SPSS ver. 23.0 (SPSS, Chicago, IL, USA). Numerical data were expressed as medians and ranges. Categorical data were described using numbers and percentages. OS and PFS was determined using the Kaplan–Meier method and compared with patients receiving different treatments. Cox proportional hazards regression analysis of mortality and recurrence in HCC patients was performed. Furthermore, we used logistic regression to generate propensity score matching (PSM) with age, sex, cirrhosis, CP class A5 and antiviral therapy to reduce bias in our analyses. The two treatment groups were matched with the control group according to PSM using a caliper width of 0.02. After PSM, the baseline covariates were compared using the paired *t* test or Mann–Whitney U test for continuous variables and the chi‐square test for categorical variables. A *p* value of <0.05 indicated statistical significance.

## 3 RESULTS

### 3.1 Baseline characteristics

The demographic and clinical features of the 298 HCC patients (79.2% male, median age of 64 years) with single and large (≥5 cm) tumors with CP class A, but without distant metastasis or/and macrovascular invasion are shown in Table 1. Regarding the etiology of HCC, 44.1% of the patients had HBV infection, 23.2% had HCV infection, and 30.6% had non‐HBV and HCV. Approximately 34.6% of patients had liver cirrhosis, and 89.3% had CP class A5 disease. The initial treatment modality was SR (50.8%) and TACE (49.2%), respectively. The median follow‐up time was 33 months.

**TABLE 1:** Basic demographic and clinical characteristics of the included patients

Variables	Total patients	Resection (*n* = 151)	TACE (*n* = 147)	*p*‐value
Demographic variables				
Age (years)	64 (23–92)	61 (23–88)	66 (25–92)	<0.001
Sex: Male	236 (79.2)	120 (79.5)	116 (78.9)	0.905
BMI (kg/m^2^)	25.0 (16.0–38.9)	25.0 (16.0–36.8)	25.0 (18.2–38.9)	0.998
Diabetes	49 (16.4)	30 (19.9)	19 (12.9)	0.106
Hypertension	60 (20.1)	43 (28.5)	17 (11.6)	<0.001
Smoking	84 (28.2)	35 (23.2)	49 (33.3)	0.051
Alcohol use	54 (18.1)	22 (14.6)	32 (21.8)	0.107
Etiology				
Non‐B Non‐C	91 (30.6)	41 (27.2)	50 (34.2)	0.062
HBV positive	131 (44.1)	79 (52.3)	52 (35.6)	
HCV positive	69 (23.2)	29 (19.2)	40 (27.4)	
Both HBV and HCV positive	6 (2.0)	2 (1.3)	4 (2.7)	
Cirrhosis	103 (34.6)	38 (25.2)	65 (44.2)	0.001
Child‐Pugh class A5	266(89.3)	145 (96.0)	121 (82.3)	<0.001
Antiviral therapy	96 (32.2)	40 (26.5)	56 (38.3)	0.032
Laboratory variable				
Platelet count (10^9^/L)	173 (62–401)	178 (64–401)	169 (62–401)	0.240
Total bilirubin (mg/dl)	0.9 (0.1–2.9)	1.0 (0.1–2.5)	0.9 (0.1–2.9)	0.079
Serum albumin (g/dL)	4.1 (3.1–4.8)	4.1 (3.2–4.8)	4.0 (3.1–4.8)	0.088
AST (IU/L)	53 (16–259)	57 (16–233)	51 (16–259)	0.173
ALT (IU/L)	54 (13–403)	56 (13–363)	50 (13–403)	0.298
INR	1.1 (0.9–2.8)	1.1 (0.9–2.0)	1.1 (0.9–2.8)	0.312
Creatinine (mg/dl)	1.2 (0.5–8.2)	1.2 (0.5–7.8)	1.2 (0.5–8.2)	0.235
Alpha‐fetoprotein (ng/ml)	1138 (2–101,685)	1415 (2–101,685)	889 (2–86,351)	0.533
Tumor variable				
Maximum tumor size (cm)	7.6 (5.0–18.4)	7.8 (5.0–18.4)	7.3 (5.0–17.8)	0.146

*Note*: Data are shown as number (%) or median (range).

Abbreviations: ALT, alanine aminotransferase; AST, aspartate transaminase; BMI, body mass index; CI, confidence interval; HBV, hepatitis B virus; HCV, hepatitis C virus; INR, international normalized ratio; TACE, transarterial chemoembolization.

### 3.2 Overall survival and progression‐free survival

**FIGURE 2:**
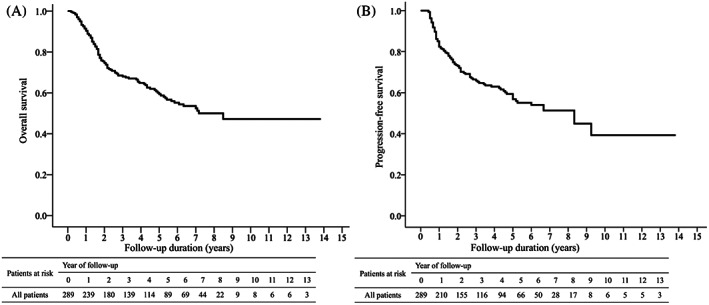
Overall survival and progression‐free survival of total cohort. The cumulative incidence of overall survival (A) and progression‐free survival (B) of total cohort.

**FIGURE 3:**
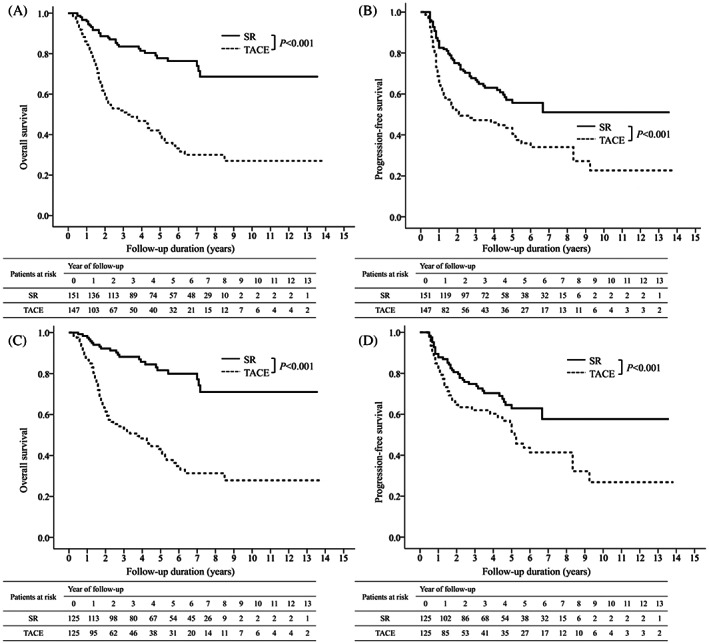
Overall survival and progression‐free survival in different treatment before and after propensity score matching. The cumulative incidence of overall survival (A) and progression‐free survival (B) in different treatment. Surgical resection resulted in significantly higher overall survival and progression‐free survival rates than TACE. After PSM, the cumulative incidence of overall survival (C) and progression‐free survival (D) in different treatment. Surgical resection resulted in significantly higher overall survival and progression‐free survival rates than TACE.

Kaplan–Meier analysis showed that the 1‐, 5‐, and 10‐year OS rates after initial treatment were 90.4%, 59.4%, and 47.2% respectively (Figure 2A), and the 1‐, 5‐, and 10‐year PFS rates were 82.4%, 56.8%, and 39.3%, respectively (Figure 2B). The OS and PFS rates were significantly higher in patients receiving SR than those receiving TACE. The 1‐, 5‐, and 10‐year OS rates were 96%, 77.8%, and 68.3% after SR and 84.5%, 39.6%, and 27.0% after TACE, respectively (*p* < 0.001, Figure 3A). The 1‐, 5‐, and 10‐year PFS rates were 82.5%, 57.1%, and 51.0% after SR and 64.3%, 40.5%, and 22.7% after TACE, respectively (*p* < 0.001, Figure 3B).

### 3.3 Overall survival and progression‐free survival after propensity score matching

After PSM, the total number of patients was 250, with 125 patients in the SR group and 125 patients in the TACE group. None of the clinical features were significantly different between the SR and TACE groups (Table S1). OS was significantly higher in the SR group than in the TACE group (*p* < 0.001) (Figure 3C). The 1‐, 5‐, and 10‐year cumulative OS rates were 97.5%, 81.6%, and 71% in the SR group and 86.8%, 41.8%, and 27.8%in the TACE group, respectively (Figure 3C). Furthermore, PFS was significantly higher in the SR group than in the TACE group (*p* < 0.001) (Figure 3D). The 1‐, 5‐, and 10‐year cumulative PFS rates were 87.8%, 62.9%, and 57.7% in the SR group and 82.1%, 51.4%, and 26.8% in the TACE group, respectively (Figure 3D).

### 3.4 Factors affecting mortality and recurrence

Diabetes, cirrhosis, CP class A5/6, AFP ≥200, tumor size ≥10 cm, and initial treatment were significantly associated with mortality in univariate analysis (Table 2). Furthermore, in multivariate analysis, cirrhosis (absent vs. present; HR: 2.04; 95% CI: 1.35–3.03, *p* < 0.001), CP class A5/6 (A5 vs. A6; HR: 4.01; 95% CI: 2.43–6.66, *p* < 0.001), and initial treatment (SR vs. TACE HR = 3.23; 95% CI: 2.13–5.01, *p* < 0.001) remained significantly associated with mortality (Table 2). Moreover, in multivariate analysis, CP class A5/6 (A5 vs. A6; HR: 3.23; 95% CI: 1.89–5.88, *p* < 0.001), and initial treatment (SR vs. TACE; HR = 4.17; 95% CI: 1.64–8.33, *p* = 0.039) remained significantly associated with recurrence (Table 2).

**TABLE 2:** Univariate and multivariate analyses of factors associated with mortality and recurrence

Demographic variables	*N* = 298	Mortality			Recurrence		
		Univariate	Multivariate		Univariate	Multivariate	
		*p*‐value	HR (95% CI)	*p*‐value	*p*‐value	HR (95% CI)	*p*‐value
Age (years)							
<60	97	1			1		
≥60	201	0.137			0.414		
Gender							
Female	62	1			1		
Male	236	0.577			0.501		
BMI (kg/m^2^)							
<25	169	1			1		
≥25	129	0.174			0.868		
Diabetes							
Absent	249	1			1		
Present	49	0.049	1.82 (0.99–3.33)	0.053	0.604		
Hypertension							
Absent	238	1			1		
Present	60	0.066			0.870		
Smoking							
Absent	214	1			1		
Present	84	0.999			0.166		
Alcohol use							
Absent	244	1			1		
Present	54	0.772			0.848		
Etiology							
Non‐B Non‐C	91	1			1		
HBV positive	131	0.159			0.259		
HCV positive	69	0.916			0.713		
HBV + HCV	6	0.652			0.198		
Cirrhosis							
Absent	195	1			1		
Present	103	<0.001	2.04 (1.35–3.03)	<0.001	0.308		
Child‐Pugh class							
A5	266	1	1		1	1	
A6	32	<0.001	4.01 (2.43–6.66)	<0.001	<0.001	3.23 (1.89–5.88)	<0.001
Antiviral therapy							
Absent	202	1			1		
Present	96	0.052			0.052		
Laboratory variable							
Platelet count (10^9^/L)							
<150	128	1			1		
≥150	170	0.762			0.633		
Total bilirubin (mg/dl)							
<1.2	231	1			1		
≥1.2	67	0.938			0.153		
Serum albumin (g/dl)							
<3.5	14	1			1		
≥3.5	284	0.637			0.558		
AST (IU/L)							
<50	175	1			1		
≥50	123	0.687			0.094		
ALT (IU/L)							
<50	195	1			1		
≥50	103	0.797			0.127		
INR							
<1.2	275	1			1		
≥1.2	23	0.259			0.090		
Creatine (mg/dl)							
<1.1	101	1			1		
≥1.1	197	0.278			0.405		
Alpha‐fetoprotein (ng/ml)							
<200	252	1	1		1		
≥200	46	<0.001	1.96 (0.84–2.04)	0.061	0.244		
Tumor variable							
Maximum tumor size (cm)							
<10	251	1	1		1		
≥10	47	0.016	0.63 (0.33–1.20)	0.162	0.341		
Treatment variable							
Initial treatment							
Resection	151	1	1		1	1	
TACE	147	<0.001	3.23 (2.13–5.01)	<0.001	<0.001	4.17 (1.64–8.33)	0.039

Abbreviations: ALT, alanine aminotransferase; AST, aspartate transaminase; BMI, body mass index; CI, confidence interval; HBV, hepatitis B virus; HCV, hepatitis C virus; HR, Hazard ratio; INR, international normalized ratio; TACE, transarterial chemoembolization.

### 3.5 Overall survival in subgroup analysis

**FIGURE 4:**
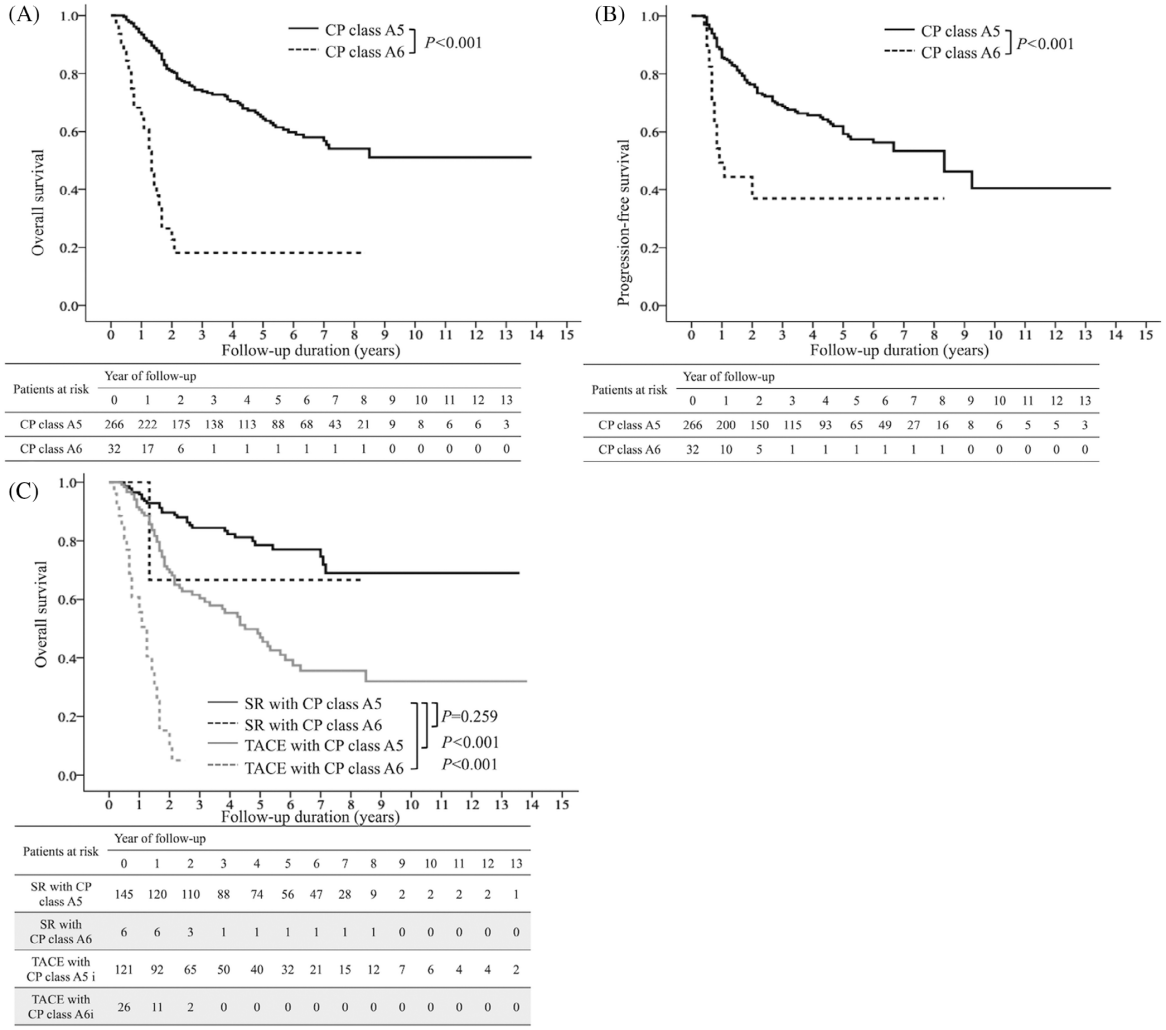
Overall survival and progression‐free survival in subgroup analysis. The cumulative incidence of overall survival (A) and progression‐free survival (B) in Child‐Pugh (CP) class A5/6. The overall survival and progression‐free survival rates were significantly higher in patients with CP class A5 than those with CP class A6. The cumulative incidence of overall survival in patients with treatment modality and CP class A (C). The overall survival was significantly higher in patients receiving SR with CP class A5 than those receiving TACE with CP class A5 or A6.

The OS and PFS rates were significantly higher in patients with CP class A5 than those with CP class A6 (*p* < 0.001, Figure 4A,B). When performing subgroup analysis limited to patients with CP class A5/6, the OS rates were significantly higher in patients receiving SR with CP class A5 than those receiving TACE with CP class A5 or A6 (*p* < 0.001, Figure 4C).

## 4 DISCUSSION

Our study showed that the OS and PFS rates were significantly higher in patients receiving SR that those receiving TACE. The 10‐year OS rates after SR and TACE were 68.3% and 27.0%, respectively. The 10‐year PFS rates after SR and TACE were 51.0% and 22.7%, respectively. CP class A5/6 and initial treatment were significantly associated with mortality and recurrence in the multivariate analysis. SR is safe and effective treatment for single large HCC.

SR is widely used as the first‐line therapy in HCC patients with good persevered liver function and tumor factors.^19^, ^20^ Previous studies that SR in Child A patients had good outcome, with 5‐year OS of over 60% and 5‐year PFS of over 40%.^12^, ^21^, ^22^, ^23^, ^24^ Our study demonstrated that 151 (50.8%) patients underwent SR, and showed better 5‐year survival (77.8%) and progression‐free survival (39.6%) rates than those receiving TACE. This is consistent with previous studies that demonstrated comparable prognosis in large HCC patients and proposed considering hepatectomy not based on tumor size.^6^, ^11^ Previous studies reported that patients receiving SR had better OS rates than those receiving TACE in BCLC stage B HCC patients.^8^, ^25^ Recent, several studies showed survival benefit of liver resection for BCLC stage B HCC patients.^16^, ^17^, ^23^, ^24^, ^26^, ^27^, ^28^, ^29^ Moreover, our study is also consistent with previous study demonstrating that SR was significantly associated with better OS and PFS than TACE.^12^

CP class was significantly associated with OS in HCC patients in different BCLC stage receiving different treatment.^3^, ^4^, ^5^ Our study showed that CP class A5 were significantly associated with better OS and PFS in single large HCC patients in multivariate analysis. Our results were different from previous study showing there is no significant difference on OS and PFS between CP class A5 and A6 in single large HCC patients in multivariate analysis.^12^ To the best of our knowledge, our study was the first to point out that CP class A5 is significantly associated with mortality and recurrence in single large HCC patients.

The limitations of the study include the following. First, the retrospective nature of the study might have resulted in unintended bias. Second, PFS may be biased especial in the patients receiving non‐curative therapies. Third, the study is the long recruitment period which means including patients according to different guidance with time.

In conclusion, SR had significantly better OS and PFS rates than TACE. CP class A5/6 and initial treatment were significantly associated with mortality and recurrence. SR is an effective and safe therapy for single large HCC.

## CONFLICT OF INTEREST

All authors declare no conflict of interest.

## Supporting information


**Table S1:** Basic demographic and clinical characteristics after propensity scoring matching.
