# Supplementary material for: Clinical prognosis of surgical resection versus transarterial chemoembolization for single large hepatocellular carcinoma (≥5 cm): A propensity score matching analysis
Source: Kaohsiung J Med Sci. 2023 Jan 10;39(3):302–10. doi: 10.1002/kjm2.12640 (PMC11895981; doi:10.1002/kjm2.12640)
Supplement: Supplementary file 1 — Table S1: Basic demographic and clinical characteristics after propensity scoring matching. [file KJM2-39-302-s001.docx]

Table S1: Basic demographic and clinical characteristics after propensity scoring matching

| **Variables** | Resection (n=125) | TACE (n=125) | *P*-value |
| --- | --- | --- | --- |
| **Demographic variables** |  |  |  |
| Age (years) | 63 (23-88) | 64 (25-92) | 0.063 |
| Sex: Male | 96 (76.8) | 98 (78.4) | 0.762 |
| BMI (kg/m^2^) | 25.0 (16.0-36.8) | 25.0 (18.2-38.9) | 0.972 |
| Diabetes | 25 (20.0) | 17 (13.6) | 0.176 |
| Hypertension | 26 (20.8) | 16 (12.8) | 0.083 |
| Smoking | 29 (23.2) | 41 (32.8) | 0.061 |
| Alcohol use | 20 (16.0) | 28 (22.4) | 0.194 |
| Etiology |  |  |  |
| Non-B Non-C | 32 (25.6) | 39 (31.5) | 0.071 |
| HBV positive | 67 (53.6) | 45 (36.3) |  |
| HCV positive | 24 (19.2) | 36 (29.0) |  |
| HBV+HCV positive | 2 (1.6) | 4 (3.2) |  |
| Cirrhosis | 33 (26.4) | 45 (36.0) | 0.053 |
| Child-Pugh class A5 | 120 (96.0) | 111 (88.8) | 0.098 |
| Antiviral therapy | 35 (28.0) | 46 (36.8) | 0.139 |
| **Laboratory variable** |  |  |  |
| Platelet count (10^9^/L) | 175 (64-388) | 172 (62-401) | 0.211 |
| Total Bilirubin (mg/dL) | 0.9 (0.1-2.3) | 0.9 (0.1-2.6) | 0.195 |
| Serum albumin (g/dL) | 4.1 (3.3-4.8) | 4.0 (3.3-4.8) | 0.203 |
| AST (IU/L) | 55 (16-233) | 52 (16-259) | 0.644 |
| ALT (IU/L) | 56 (13-363) | 50 (13-403) | 0.666 |
| INR | 1.1 (0.9-2.0) | 1.1 (0.9-2.8) | 0.742 |
| Creatinine (mg/dL) | 1.2 (0.5-7.8) | 1.3 (0.5-8.2) | 0.123 |
| Alpha-fetoprotein (ng/mL) | 1255 (2-91628) | 958 (2-86351) | 0.462 |
| **Tumor variable** |  |  |  |
| Maximum tumor size (cm) | 7.6 (5.0-18.1) | 7.4 (5.0-17.8) | 0.359 |

# Data are shown as number (%) or median (range). BMI: Body mass index; HBV: Hepatitis B virus; HCV: Hepatitis C virus; AST: Aspartate Transaminase; ALT: Alanine aminotransferase; INR: International Normalized Ratio; TACE: Transarterial chemoembolization;
